# Biochar-extracted liquor stimulates nitrogen related gene expression on improving nitrogen utilization in rice seedling

**DOI:** 10.3389/fpls.2023.1131937

**Published:** 2023-06-19

**Authors:** Jian Gao, Shaohua Ge, Hailong Wang, Yunying Fang, Luming Sun, Tianyi He, Xiaoyi Cheng, Di Wang, Xuanwei Zhou, Heqing Cai, Caibin Li, Yanxiang Liu, Yang E, Jun Meng, Wenfu Chen

**Affiliations:** ^1^ National Biochar Institute of Shenyang Agricultural University, Shenyang, China; ^2^ Key Laboratory of Biochar and Soil Improvement, Ministry of Agriculture and Rural Affairs, Shenyang, China; ^3^ School of Environmental and Chemical Engineering, Foshan University, Foshan, China; ^4^ Australian Rivers Institute, School of Environment and Science, Griffith University, Nathan, QLD, Australia; ^5^ Bijie Tobacco Company of Guizhou Province, Bijie, China

**Keywords:** biochar-extracted liquor, molecular docking, nitrogen metabolism, OsAMT1.1 protein, rice seedlings

## Abstract

**Introduction:**

Biochar has been shown to be an effective soil amendment for promoting plant growth and improving nitrogen (N) utilization. However, the physiological and molecular mechanisms behind such stimulation remain unclear.

**Methods:**

In this study, we investigated whether biochar-extracted liquor including 21 organic molecules enhance the nitrogen use efficiency (NUE) of rice plants using two N forms (NH_4_^+^-N and NO_3_^-^-N). A hydroponic experiment was conducted, and biochar-extracted liquor (between 1 and 3% by weight) was applied to rice seedlings.

**Results:**

The results showed that biochar-extracted liquor significantly improved phenotypic and physiological traits of rice seedlings. Biochar-extracted liquor dramatically upregulated the expression of rice N metabolism-related genes such as *OsAMT1.1*, *OsGS1.1*, and *OsGS2*. Rice seedlings preferentially absorbed NH_4_^+^-N than NO_3_^-^-N (*p* < 0.05), and the uptake of NH_4_^+^-N by rice seedlings was significantly increased by 33.60% under the treatment of biochar-extracted liquor. The results from molecular docking showed that OsAMT1.1protein can theoretically interact with 2-Acetyl-5-methylfuran, trans-2,4-Dimethylthiane, S, S-dioxide, 2,2-Diethylacetamide, and 1,2-Dimethylaziridine in the biochar-extracted liquor. These four organic compounds have similar biological function as the OsAMT1.1 protein ligand in driving NH_4_^+^-N uptakes by rice plants.

**Discussion:**

This study highlights the importance of biochar-extracted liquor in promoting plant growth and NUE. The use of low doses of biochar-extracted liquor could be an important way to reduce N input in order to achieve the purpose of reducing fertilizer use and increasing efficiency in agricultural production.

## 1 Introduction

Nitrogen (N) is a limiting nutrient element for plants and is crucial for crop productivity in agriculture (Vitousek et al., 2002). In recent decades, N demand for crop productivity has sharply increased in order to maintain maximal crop yield, as only 30-50% of the applied N fertilizer can be absorbed by crops (Pathak et al., 2008; McAllister et al., 2012; Anas et al., 2020; Einarsson et al., 2021). The excessive application of N fertilizer has resulted in soil acidification (Tian and Niu, 2015; Yang, 2019), hardening, low nitrogen use efficiency (NUE) of plant (Xia et al., 2022). Enhancing crop production without increasing N fertilizer application, such as improving NUE, is an urgent challenge in agriculture. In doing so, some technologies have been developed, including slow-release N fertilizer (Tian et al., 2020), organic fertilizer (Liu et al., 2017), straw carbonization returning (biochar) (Khan et al., 2020). The pyrolysis process of biomass is prone to making the organic N into inorganic N (Xiao and Chen, 2017), which is easily absorbed by plants, further promoting plant growth. A meta-analysis showed that biochar application significantly increased plant N uptake (Liu et al., 2018), improved plant yield and NUE. Changes in the interaction between mineral N and hormones caused by biochar may influence N assimilation (Feng et al., 2021; Javed et al., 2022). It is suggested that biochar changes during root development, which may contribute to the N cycle, in particularly by helping to increase the opportunity to capture N from fertilizer and soil (E et al., 2019; Martínez-Gómez et al., 2022). However, there is still insufficient evidence to support that biochar affects N metabolism *via* inducing changes in nitrogen genotypes.

Biochar is a new and old term (Chen et al., 2019) and refers to a carbon-rich solid product that forms when biomass is thermally decomposed under anoxic conditions (Lehmann et al., 2006). Because of its unique physical and chemical properties, biochar can not only be used as a soil amendment for improving soil quality but also as a promising carrier for slow-release fertilizers (Hossain et al., 2020; Phillips et al., 2022). Biochar can not only promote N absorption, but also promote N assimilation by regulating N related physiological metabolism processes (Feng et al., 2021; Javed et al., 2022). There are some reports suggested that the C/N ratio, pH and ash content of biochar affect N adsorption by plant root and NUE (Cai et al., 2016; Ibrahim et al., 2020). Recent studies had shown that biochar-extract liquor as a DOM (dissolvable organic matter) fraction had been known of a composition of organic compounds as biopolymers, humics, building blocks, low molecular weight acids, and low molecular weight neutrals as well as hydrophobic organic carbon (Lou et al., 2015; Bian et al., 2019). Further, labile organic molecules from fast pyrolysis of rice husk are beneficial to improve plant metabolic processes, which directly affect the related gene and protein expression (Yuan et al., 2017; E et al., 2019; Yang et al., 2022). Several organic matters of biochar including 2-Acetyl-5-methylfuran have been reported to affect the plant growth and development (E et al., 2019). However, there are few studies on NUE relating to biochar organic compounds. Understanding how biochar organic compounds affect NUE may be the key factor for determining the optimized biochar application.

It is generally accepted that three key intrinsic factors affect plant NUE, including N uptake, assimilation, and efficient remobilization (Xu et al., 2012; Chen et al., 2020). Nitrogen (in the forms of ${\text{NH}}_{4}{\;}^{+}-\text{N}$ , ${\text{NO}}_{3}{\;}^{-}-\text{N}$ , and other organic molecules) can be assimilated in roots or be transported to shoots. ${\text{NH}}_{4}{\;}^{+}-\text{N}$ and ${\text{NO}}_{3}{\;}^{-}-\text{N}$ are absorbed and transported *via* different pathways. The uptake of ${\text{NH}}_{4}{\;}^{+}-\text{N}$ involves a group of high-affinity transport system (HATS) transporters known as ammonium transporters (AMTs) (Naesholm et al., 1998). ${\text{NH}}_{4}{\;}^{+}-\text{N}$ is directly assimilated after being transported into the cells. ${\text{NH}}_{4}{\;}^{+}-\text{N}$ is further used in the production of nitrogenous compounds catalyzed by GS/GOGAT cycle (glutamine synthetase/glutamate synthase). ${\text{NO}}_{3}{\;}^{-}-\text{N}$ is absorbed by plant cells through nitrate transporters (NRTs) that includes HATS and/or low-affinity transport systems (LATS) (Dechorgnat et al., 2011; Anne et al., 2014; Xuan et al., 2017). Unlike AMTs, which only transport ${\text{NH}}_{4}{\;}^{+}-\text{N}$ , a well-characterized NRT, NRT1.1 is a ${\text{NO}}_{3}{\;}^{-}-\text{N}$ symporter that mediates the uptake of both ${\text{NO}}_{3}{\;}^{-}-\text{N}$ and protons (H^+^). Before assimilation, absorbed ${\text{NO}}_{3}{\;}^{-}-\text{N}$ must be catalyzed by nitrate reductase (NR) and nitrite reductase (NiR) to form ${\text{NH}}_{4}{\;}^{+}-\text{N}$ (Xu et al., 2012). Hence, the N-related gene expression plays a crucial role in NUE. Studies have shown that *OsAMT1.1*, *OsNR2*, *OsNPL3* and *OsNPL4* are important genes in the N metabolic pathway in rice (Ranathunge et al., 2014; Li et al., 2016; Gao et al., 2019; Hu et al., 2019) when the N is absorbed from exogenous N. So, the genes are the mark for nitrate uptake. Biochar organic compounds was found to regulate plant gene expression (Yuan et al., 2017; E et al., 2020). However, little is known about the role of organic compounds in biochar on promoting N-related gene expression and the associated plant NUE. Besides, biochar contains a large number of organic compounds, which make it difficult in identifying those individuals that enhance NUE. The molecular docking, originated from the key-locking principle (Koshland, 1958; Richard, 1995; Tripathi and Bankaitis, 2017), may be able to link the biological functions of particular biochar organic compounds with plant NUE. This principle refers to the process of mutual recognition between a ligand (e.g., a small molecule) and a receptor (e.g., a protein) through geometric and energy matching (Chen et al., 2006). Specifically, a ligand can bind to a receptor, forming ligand-receptor complexes that perform relevant biological functions. It is considered that the exogenous small molecules including biochar organic compounds have the same function as the protein ligand if they can also bind to the active site of the receptor (e.g., the target protein) and have complementary spatial conformation and energy. As such, the method of molecular docking is feasible to study the virtual biological activity of biochar affecting plant N utilization (E et al., 2019).

Motivated by the previous studies, we hypothesized that the organic compounds from biochar would have important effect on expression of N-related genes in rice plant, further improving NUE. The main objectives of this study were (1) to quantify the effects of the biochar-extracted liquor on NUE using different inorganic N forms, (2) to determine the genes expression in N metabolism during biochar-extracted liquor treatment and (3) to reveal the functional role of organic molecules on the surface of biochar in biochar-induced N metabolism in rice seedlings.

## 2 Materials and methods

### 2.1 Preparation of biochar-extracted liquor

The raw materials (including tobacco straw, broomcorn straw, maize straw, soybean straw, peanut straw, rice straw and rice husk) used in this study were obtained from the Rice Research Institute of Shenyang Agricultural University in China. All materials were washed in Milli-Q ultrapure water (Millipore company, USA) for 5 min to remove dust, and were air-dried before grinding to < 50 mesh sieves size (280 μm). The powders of the materials (including rice husk, rice straw, peanut straw, tobacco straw, soybean straw, maize straw and sorghum straw) were pyrolyzed under oxygen-limited conditions at 300°C, 400°C, 500°C, 600°C and 700°Cwith a heating rate of 5°C/min holding for 30 min, separately. The biochar-extracted liquor was prepared at 0%, and 1%, 3%, and 5% concentrations (biochar: water = w: w). The different concentrations were mainly produced by washing the corresponding dry weight of the biochar with ultrapure water. After shaking at 25 °C for 24 h, the biochar-extracted liquor was filtered through a 0.22 μm filter, sterilized at 121 °C for 60 min in order to eliminate the effect of microorganism, and stored at 4 °C (Yuan et al., 2017). Among these 35 biochars, the rice husks biochar pyrolyzed at 400°C was the best on enhancing NUE in the preliminary screening experiment. Therefore, the rate of NUE enhanced by rice husks biochar pyrolyzed at 400°C is preferred.

### 2.2 Experimental design

Rice seeds (*Japonica* super rice Shennong 265) were disinfected (15% NaClO for 15min), soaked in sterilized water for 48 h. Rice seeds are placed in petri dishes and placed in a greenhouse (30°C in the dark) for germination. After germination, rice seedlings were grown hydroponically in 96 well-plates with different N forms ( ${\text{NH}}_{4}{\;}^{+}-\text{N}$ and ${\text{NO}}_{3}{\;}^{-}-\text{N}$ ), which were established treatments of 1:0 and 0:1 ${\text{NH}}_{4}{\;}^{+}-\text{N}$ : ${\text{NO}}_{3}{\;}^{-}-\text{N}$ in a modified Kimura B nutrient solution (Ho et al., 2018; Wang et al., 2018). Each treatment was repeated three times. Except for the different N forms, the contents of the other nutrient elements were the same. The modified Kimura B solution contained the following macronutrients: 0.54 mM MgSO_4_·7H_2_O, 0.36 mM CaCl_2_, 0.09 mM K_2_SO_4_, 0.18 mM KH_2_PO_4_ and 1.6 mM Na_2_SiO_3_·9H_2_O; and micronutrients: 9.14 μM MnCl_2_·4H_2_O, 46.2 μM H_3_BO_3_, 0.08 μM (NH_4_)_6_Mo_7_O_24_·4H_2_O, 0.76 μM ZnSO_4_·7H_2_O, 0.32 μM CuSO_4_·5H_2_O, and 40 μM Fe(II)-EDTA, with the pH adjusted to 5.5-6.0. The total N content of each treatment solution was 2 mM. The nutrient solution was changed every three days.

**Figure 1:**
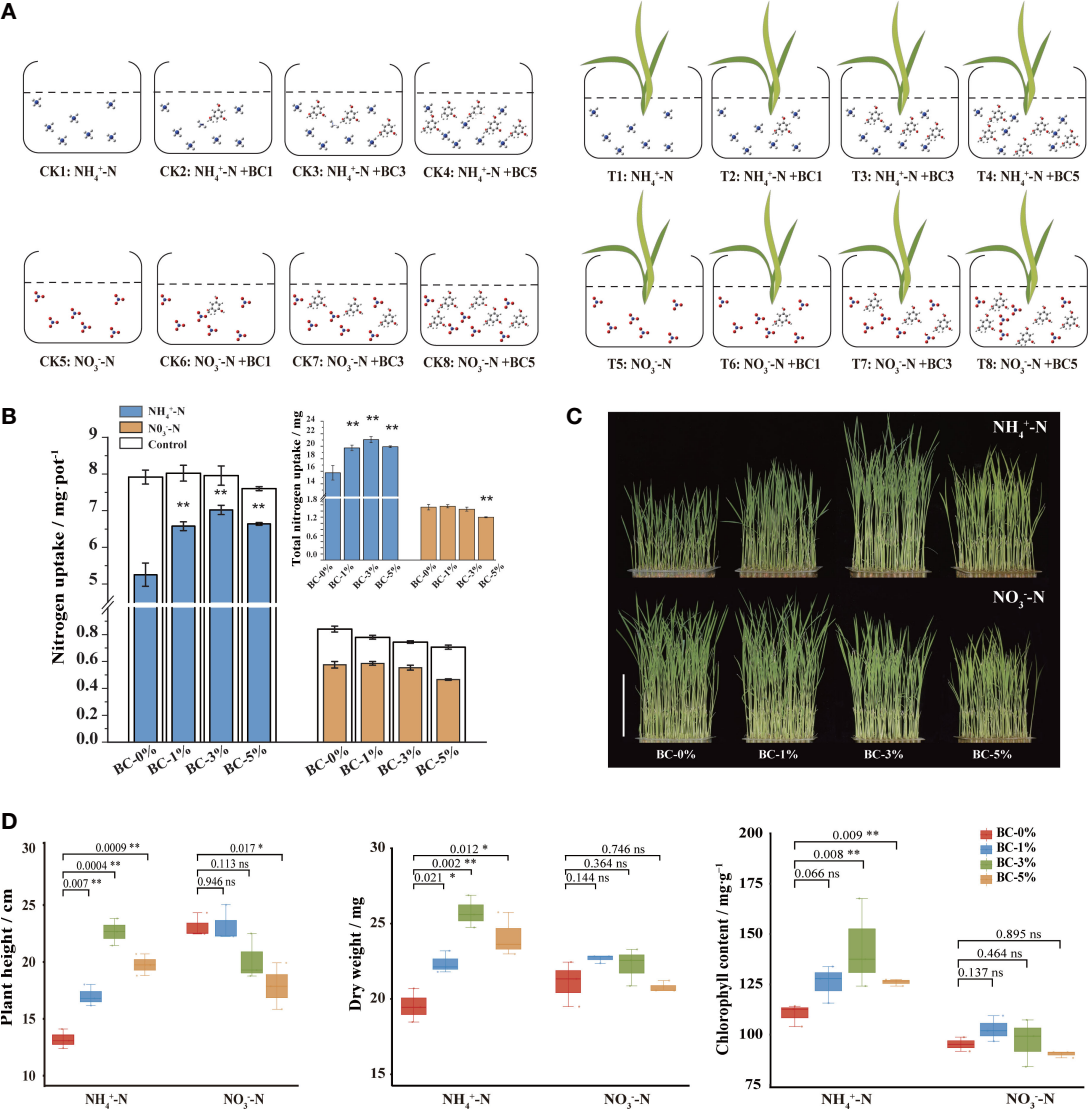
Response of rice seedlings to biochar-extracted liquor under two N forms conditions. **(A)** Diagram of the experimental design for the hydroponic experiment. Biochar-extracted liquor with different concentrations was added to a nutrient solution with ammonium or nitrate N. They were divided into the controls (CK1–8; without plants) and the treatment (T1–8; with plants) groups. The volatilization of N in the nutrient solution were detected in the control group. The N uptake by the rice seedlings were detected in the treatment group. **(B)** Response of N uptake and total uptake of rice seedlings under two N forms (ammonium N and nitrate N). **(C)** Phenotypes of rice seedlings grown in modified Kimura B, supplemented with 2 mM NH_4_^+^/NO_3_^-^ and biochar-extracted liquor extracted at different ratios of 1:100, 3:100 and 5:100, noted as 1%, 3% and 5%. **(D)** Effects of biochar on agronomic traits of rice seedlings (plant height, dry weight, chlorophyll content). The data in the graphs are presented as mean ± standard deviation, n = 3. * and ** indicate significant differences at *p* < 0.05 and *p* < 0.01, respectively.


To quantify the N uptake of rice seedlings under the biochar-extracted liquor treatment, a hydroponic experiment was designed. The nutrient solution was prepared by adding different concentrations of biochar-extracted liquor to the two N forms (ammonia and nitrate N). During rice seedling growth, the nutrient solution was regularly replaced to avoid nutrient deficiency and changes in N content in the nutrient solution was measured. The control solution was replaced at the same time. The nutrient solution was divided into two parts: one for the control (CK1-8) and the other for cultivating rice seedlings (T1-8) (**Figure 1A**). We assumed that the total N content in the hydroponic system included rice N uptake, the residual N content of the nutrient solution, and volatilized N (NO, N_2_O, NH_3_). N uptake in rice seedlings was calculated using the subtraction method:

(1) $${\text{N}}_{\text{uptake}}={\text{ N}}_{\text{total}}–{\text{ N}}_{\text{nutrient solution}}-\;{\text{N}}_{\text{volatility}}\;\;\;\;\;\;\;\;\;\;\;\;\;\;\;\;\;\;\;\;\;\;\;\;\;\;\;\;\;\;\;$$

The seedlings were grown in a growth chamber under controlled conditions (photoperiod 12 h-light and 12 h-dark at 28 or 25°C). Root and leaf parts were collected at the seedling stage (21 days). After collection, some samples were immediately frozen and stored at -80°C until enzyme assays and RNA extraction. The remaining samples were used for determining the phenotypic traits of rice seedlings. All experiments were repeated three times.

### 2.3 Identification of organic compounds extracted from biochar

According to the principle of similar compatibility, polar and non-polar organic solvents were used to extract organic compounds from biochar. A 1.5 g sample of biochar was added to 100 mL of homogeneous non-polar organic solvents (n-heptane, n-hexane) and polar organic solvents (methanol, ethanol, acetonitrile, chloroform, ethyl acetate, and dichloromethane) (Lievens et al., 2015). Then, 100 μL of polar organic solvent extracts were dried under N evaporation to identify the main compounds in the leachates of different organic solvents. In the derivatization process, 50 μL of methoxy amine hydrochloride (including 20 mg·mL^-1^ pyridine) was added as the first derivatization agent. The mixture was incubated at 60 °C for 45 min. Next, 100 μL N, O-BIS (trimethylsilyl) trifluoroacetamide (BSTFA) was added, and incubated at 60 °C for 45 min.

Chromatographic conditions: The 7890A GC-240MS was used for GC-MS (Agilent accompany, USA). The chromatographic column used was a VF-5MS (30 m × 0.25 mm). The inlet temperature was 280°C. Helium (99.999%) was used as the carrier gas at a flow rate of 1.0 mL/min and a split ratio of 75:1. The heating program was maintained at 40°C for 1 min and then increased to 295°C with a heating rate of 8°C/min for 4 min. An EI ion source with an electron bombardment capability of 70 eV was used at a temperature of 200°C. The mass spectrometer was delayed for 3 min, and the mass detection range was 10-500 m/z. The detection voltage was 1600 V.

All compounds detected by GC/MS were identified and aligned using Wiley 6.0 (Wiley, New York, NY, USA) and the Mass Spectral Library (version 2.0, National Institute of Standards and Technology, NIST/EPA/NIH, USA). The relative amount of each compound was determined by analyzing the total integrated area (1000) of each sample. Compounds were identified with a probability >80% in the library search procedure.

### 2.4 Determination of N metabolizing enzymes in rice seedlings

The activities of nitrate reductase (NR), nitrite reductase (NiR), glutamine synthetase (GS), glutamate dehydrogenase (GDH), and glutamate synthase (GOGAT) were measured using enzyme-linked immunosorbent assays (ELISAs) (Yu et al., 2021). Each treatment was repeated three times. The concentrations of NH_4_^+^ and NO_3_^-^ in the solutions were determined using an Auto Analyzer3 (Seal Analytical GmbH, Germany). After harvesting of 21-days-old rice seedlings, the samples were initially desiccated at 105°C and then oven-dried at 80°C until a stable weight. The N contents of the rice seedling leaves and roots were determined using an elemental analyzer (Vario MACRO cube, Germany). All experiments were repeated three times.

### 2.5 Real-time quantitative PCR analysis

To detect the transcriptional levels of N metabolism genes under various treatments, real-time quantitative PCR was performed using the SYBR^®^ Premix Ex Taq kit (TaKaRa) and a 7500 Real-Time PCR System (ABI, USA). The following cycling parameters were used for all PCRs: 95 °C for 30 s, 40 cycles at 95°C for 5 s, and 60 °C for 34 s. All reactions were performed at least in triplicate. *OsActin* was used as an internal control. The primer pairs used are listed in **Table S1**.

### 2.6 Molecular docking

According to molecular docking theory, N-related protein (receptor protein) structures were downloaded from the database (https://www.rcsb.org/). In total, 21 organic compounds from the biochar were identified as ligands using GC/MS. The molecular structure maps of the organic compounds from the biochar-extracted liquor were obtained using Gaussian View software. Gaussian 16 was employed for Density functional theory (DFT) calculations *via* ωB97XD functional in conjunction with def2-TZVP basis set (Tang et al., 2018; Liu et al., 2020; Lu and Chen, 2021). We also obtained stable conformations of the organic compounds extracted from the biochar. AutoDock Vina software (Trott and Olson, 2009) was used for molecular docking to simulate the interactions between the 21 organic biochar components and N-related proteins. The PyMOL software package was used to visualize the docked conformations (Delano, 2002). When both the organic components from the biochar and the ligands of N-related proteins can bind to the same domain of the active site as the proteins, the biological functions of compounds from the biochar and N-related ligands are assumed to be the same.

### 2.7 Statistical analysis

Statistical analysis was conducted using the SPSS software (version 26.0; IBM, Inc., USA). One-way analysis of variance (ANOVA) was used to test the main effects of biochar at different extraction ratio on the measured variables. When the F-statistic was significant, the means were compared using the least significant difference (LSD). Pearson’s correlation evaluated the correlation between rice seedling phenotype and N-related enzyme activities. Co-occurrence networks were visualized using the Gephi software (http://gephi.github.io/), and the results were considered to indicate a valid interactive event with Spearman correlations (*R* > 0.7, *p* < 0.05). The partial least squares (PLS) are employed to analysis how the biochar-extracted liquor affected rice N uptake by regulating the relationship between N uptake, N related enzyme activity and N related genes in rice seedlings in structural equation modeling (SEM). The estimates of path coefficients and coefficients of determination (*R* ^2^) in our path model were validated using SmartPLS 2.0 software (Ringle et al., 2005).

## 3 Results

### 3.1 Effects of biochar-extracted liquor on N uptake in rice seedlings

It has been proposed that the carbon skeleton of biochar is unable to function because the stacked structure of carbon is too large in molecular weight to enter the plant (Lehmann and Joseph, 2015). Therefore, it has no direct influence on plants. Then, it is worth thinking whether the elements (**Tables S2, S3**) and organic compounds in biochar affect the physiological and biochemical processes of plants. The N uptake of rice seedlings under biochar-extracted liquor treatment was evaluated by measuring the changes in the N content in the nutrient solution (**Figure 1A**). The results showed that the addition of biochar-extracted liquor had different effects on the uptake of the two N forms by rice seedlings. It is possible that some components in the biochar-extracted liquor affected the different N uptake pathways in rice. The addition of biochar-extracted liquor promoted the uptake of ${\text{NH}}_{4}{\;}^{+}-\text{N}$ by rice seedlings, reaching a maximum at a concentration of 3%, which significantly increased by 33.60%. On the contrary, biochar-extracted liquor addition did not have a significant effect on the uptake of ${\text{NO}}_{3}{\;}^{-}-\text{N}$ conditions by rice seedlings (**Figure 1B**). The amount of N absorbed during seedling growth also showed the same trend. The results indicated that the biochar-extracted liquor may contain some components, which can promote the uptake of ${\text{NH}}_{4}{\;}^{+}-\text{N}$ in rice.

**Figure 2:**
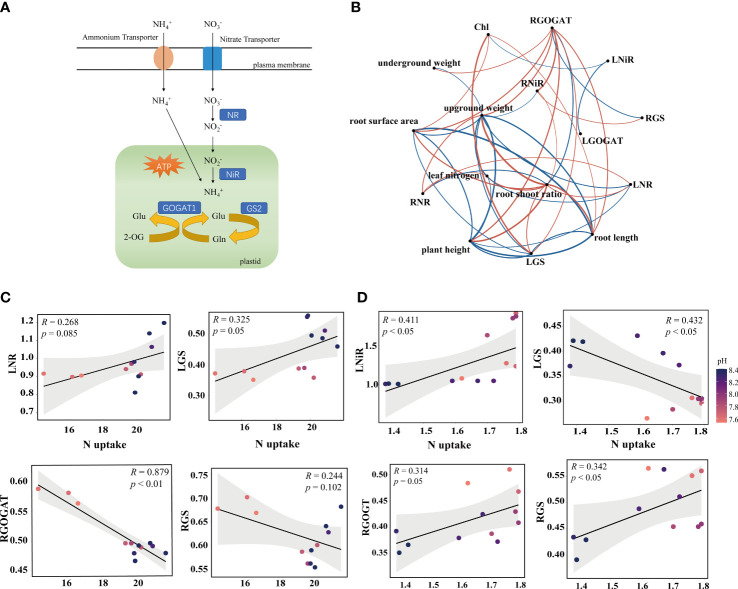
Correlation analysis of N metabolism enzymes. **(A)** Diagram of N uptake and assimilation pathway. **(B)** Co-occurrence networks between rice seedlings phenotypic traits and enzyme activity treated with ammonium N. Phenotypic traits include plant height, root length, root surface area, aboveground weight, underground weight, root shoot ratio, leaf N and chlorophyl (Chl). The N-related enzyme activity includes N metabolizing enzyme activities in the leaves such as LNR (Nitrate reductase), LNiR (Nitrite reductase), LGOGAT (Glutamate synthase), and LGS (Glutamine synthetase); and the roots such as RNiR, RGOGAT, and RGS. Red and blue arrows indicate positive and negative relationships, respectively. Co-occurrence networks between rice seedling phenotypic traits and enzyme activity treated with nitrate N are shown in **Figure S1C**. **(C, D)** Spearman’s correlation analysis of N uptake and enzyme activity in rice under ammonium N and nitrate N forms. The confidence interval (CI) is 95%.

We observed biochar-extracted liquor improved plant N uptake and utilization by increasing rice seedling height, dry weight, and chlorophyll content under ammonium-N conditions, but not under nitrate-N (**Figure 1C**). Briefly, under ammonium-N and raising the biochar-extracted liquor from 0 to 3%, plant height increased from 13 cm to 23 cm (*p* < 0.05), the dry weight increased from 0.10 g to 0.12 g (*p* < 0.05) and chlorophyll content increased from 110 to 143 mg g^-1^ (*p* < 0.05), but all the measured phenotypic traits were inhibited at the 5% biochar-extracted liquor (*p* < 0.05). Rice prefer ${\text{NH}}_{4}{\;}^{+}-\text{N}$ to ${\text{NO}}_{3}{\;}^{-}-\text{N}$ , roots can absorb ${\text{NO}}_{3}{\;}^{-}-\text{N}$ and convert the absorbed ${\text{NO}}_{3}{\;}^{-}-\text{N}$ into ${\text{NH}}_{4}{\;}^{+}-\text{N}$ for N utilization in rice. ${\text{NO}}_{3}^{‐}‐\text{N}$ is also the main N source for rice growth (**Figure 2A**). Therefore, we studied two types of N sources in the experiment. Under the same nitrate and N conditions, the effect of biochar extract on plant height and dry weight of rice was not significant (p>0.05). In a word, under the condition of ammonia N, the biochar-extracted liquor improved the N absorption and utilization of plants by increasing the height, dry weight and chlorophyll content of rice seedlings. N is a key macronutrient required for rice growth. It is also the main limiting nutrient. Therefore, enhanced N uptake might be a major factor contributing to increased biomass accumulation in rice seedlings treated with biochar-extracted liquor (**Figure 1**). These findings were consisted with previous studies reporting that biochar-extracted liquor addition augments NUE and crop yield by acting as a slow-release fertilizer and altering soil N dynamics (Huang et al., 2014; Zhang et al., 2020).

### 3.2 Response of nitrogen metabolizing enzymes to biochar-extracted liquor

N metabolizing enzymes, such as NR, GS and GOGAT play a vital role in the assimilation of N in plants (Sun et al., 2012). After absorbing inorganic N of plants, ${\text{NH}}_{4}{\;}^{+}-\text{N}$ directly enters the GOGAT cycle to produce N-containing compounds. While ${\text{NO}}_{3}{\;}^{-}-\text{N}$ is absorbed, it needs to be catalyzed by enzymes. Thus, the ${\text{NO}}_{3}{\;}^{-}-\text{N}$ utilization efficiency is lower than ${\text{NH}}_{4}{\;}^{+}-\text{N}$ in rice (**Figure 1B**). N absorbed can be transported and absorbed to roots and leaves (**Figure 2A**). The present study revealed the relationship between leaf and root enzyme activities under different N treatments (**Figure S1A**). Studies have shown that the addition of biochar increased the activity of GS, NR and GOGAT in the leaves (Farhangi-Abriz and Torabian, 2018). These changes may be due to biochar fertilization improving soil health, nutrient effectiveness and root morphological characteristics, resulting in increased N uptake and metabolism (Ali et al., 2022). Another possible explanation is the positive correlation between N accumulation in plants and N metabolizing enzymes. In present study, N content increased with LNR and RNiR under ammonium N conditions. However, under nitrate N conditions, the N content was negatively correlated with RGOGAT and RGS. In addition, the co-occurrence networks showed that ${\text{NH}}_{4}{\;}^{+}-\text{N}$ was better correlated with phenotypic traits than ${\text{NO}}_{3}^{‐}‐\text{N}$ (*R* > 0.7, *p* < 0.05) (**Figures 2B**, **S1C**). Spearman’s correlation coefficient analysis indicated that GOGAT was downregulated in the root system (*R* = 0.879, *p* < 0.01), but GS was upregulated in leaves (*R* = 0.325, *p* = 0.05) under ${\text{NH}}_{4}{\;}^{+}-\text{N}$ conditions. However, both GOGAT (*R* = 0.314, *p* = 0.05) and GS (*R* = 0.342, *p* < 0.05) were upregulated in the roots, and NiR (*R* = 0.411, *p* < 0.05) was upregulated in the leaves under ${\text{NO}}_{3}^{‐}‐\text{N}$ conditions. These results may be attributed to the different absorption paths of NH_4_^+^- and ${\text{NO}}_{3}{\;}^{-}-\text{N}$ in plants. ${\text{NH}}_{4}{\;}^{+}-\text{N}$ is transported to the leaves for assimilation to avoid ammonium toxicity (Xiao et al., 2022), whereas ${\text{NO}}_{3}^{‐}‐\text{N}$ is mainly assimilated in the roots (Liu and von Wiren, 2017; Tegeder and Masclaux-Daubresse, 2018).

### 3.3 Potential mechanisms of biochar-extracted liquor to promote nitrogen uptake

Although biochar-extracted liquor could promote N absorption and utilization in rice seedlings from the perspective of phenotype and physiology, the functional components of the biochar-extracted liquor are not known. The elements (including P, K, Na, Mg, Ca, B, Fe, and Si) contained in biochar increased with the concentration of biochar-extracted liquor. The correlation analysis of elemental content in biochar extract and N uptake by rice seedlings is shown in **Tables S3**. The results revealed that the increase of N uptake in rice seedlings was not related to the elemental content in biochar-extracted liquor (*p* > 0.05), ruling out the hypothesis that the element content of biochar impacts N uptake by rice seedlings. Thus, it can be concluded that the elements in biochar did not enhance the N uptake by rice seedlings. Previous studies have identified that the organic components of biochar have biological functions, such as promoting plant growth and resisting stress (Yuan et al., 2017; E et al., 2019). Therefore, the organic composition from biochar plays a key role in N uptake by rice seedlings (Awad et al., 2017; Liu et al., 2021; Ullah et al., 2021; Ali et al., 2022; Chew et al., 2022).

### 3.4 Screening organic molecules regulating nitrogen use efficiency

**Figure 3:**
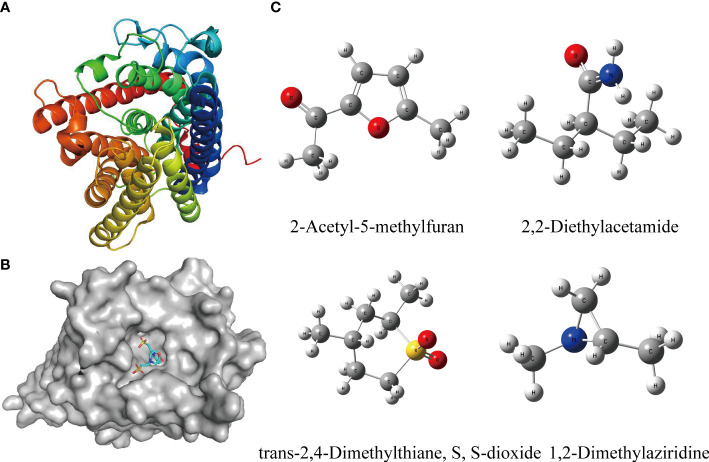
Structures of target proteins (receptors) and targeted small molecules (ligands) for molecular docking. **(A)** The 3D structure of the receptor protein (OsAMT1.1 protein) from PDB data. **(B)** The domain of active sites for interacting between receptor protein (OsAMT1.1 protein) and its ligands. **(C)** Structure of organic molecules from biochar (ligands) which can dock with the OsAMT1.1 protein. The 3D structure was built from Gaussian View. Gaussian 16 was employed for DFT calculations *via* ωB97XD functional in conjunction with the def2-TZVP basis set.

**Figure 4:**
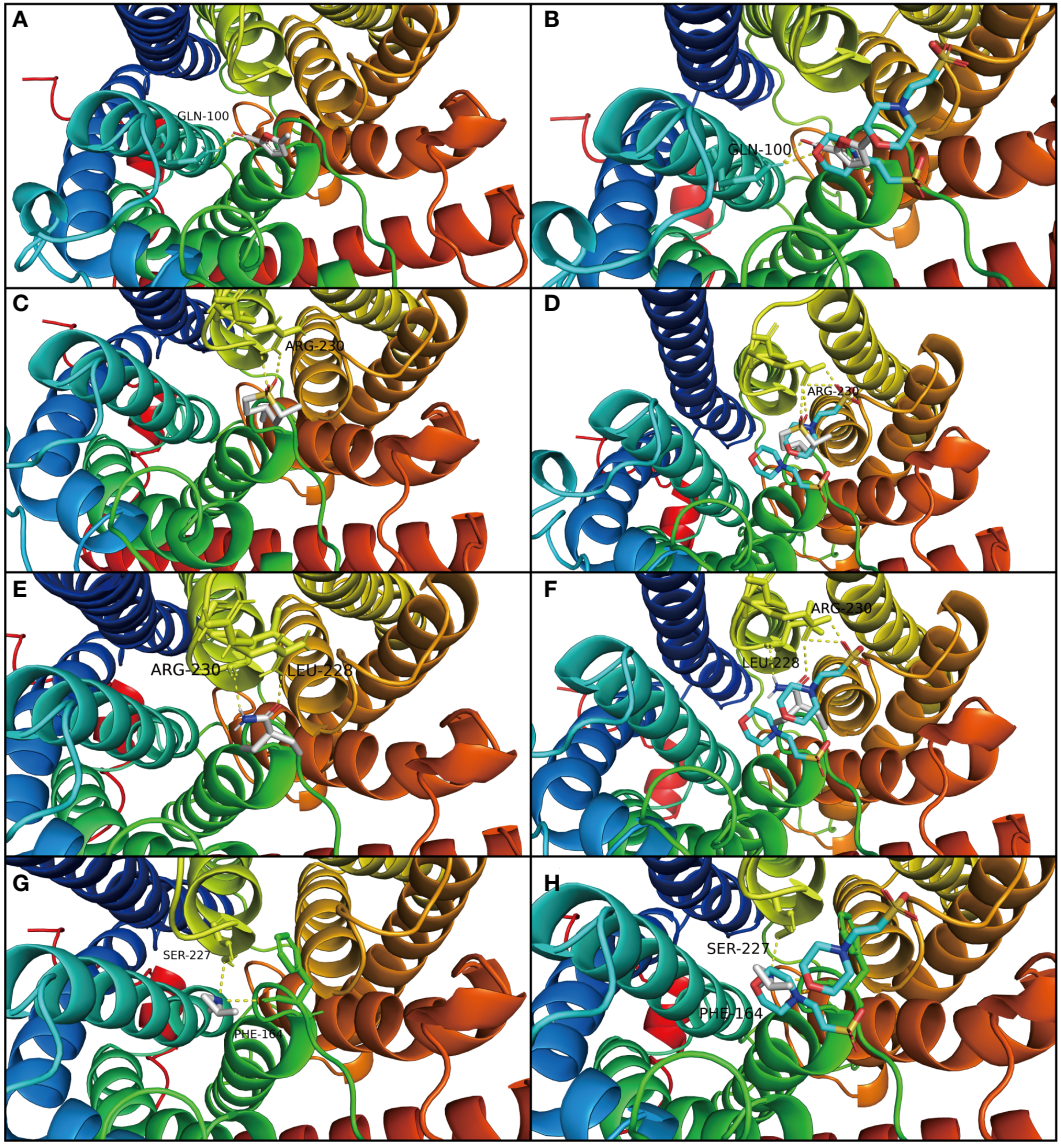
Molecular docking analysis of OsAMT1.1 protein. The panels on the left **(A, C, E, G)** show the molecule docking of the four organic molecules from biochar with AMT protein, and the panels on the right **(B, D, F, H)** show that the four biochar organic molecules (like ligand MES) have the same domain of active sites with OsAMT1.1 protein. **(A)** 2-Acetyl-5-methylfuran can connect with GLN-100 from the protein residues forming hydrogen bonds. **(B)** 2-Acetyl-5-methylfuran has the same binding sites as ligand MES such as GLN-100. **(C)** trans-2,4-Dimethylthiane, S, S-dioxide can link with the protein residues such as ARG-230, through hydrogen bonds. **(D)** trans-2,4-Dimethylthiane, S, S-dioxide has the same binding sites as ligand MES such as ARG-230. **(E)** 2,2-Diethylacetamide can link with the protein residues by hydrogen bond such as ARG-230, LEU-228. **(F)** 2,2-Diethylacetamide has the same binding sites as ligand MES such as ARG-230, LEU-228. **(G)** 1,2-Dimethylaziridine can link with SER-227, PHE-164, from protein residues by hydrogen bond. **(H)** 1,2-Dimethylaziridine has the same binding sites as ligand MES, such as SER-227, PHE-164.

The result of molecular docking suggested that the ammonium transporter protein (OsAMT1.1) successfully docked with four organic molecules from biochar (i.e., 2-Acetyl-5-methylfuran, trans-2,4-Dimethylthiane, S, S-dioxide, 2,2-Diethylacetamide, and 1,2-Dimethylaziridine) (**Figures 3A, B**). The OsAMT1.1 protein binds to the MES ligand at sites GLN-100, LYS-157, PHE-164, TYR-345, ASP-163, ASN-285, and ARG-230 through hydrogen bonds. We found that the four organic molecules of biochar can bind to the same domain of the active site (like ligand) and form hydrogen bonds with proteins, as well as form additional hydrogen bonds (**Figures 4A–H**). That is, 2-Acetyl-5-methylfuran can connect with GLN-100 from protein residues forming hydrogen bonds (**Figure 4A**); trans-2,4-Dimethylthiane, S, S-dioxide can link with protein residues, such as ARG-230 by hydrogen bonding (**Figure 4C**); 2,2-Diethylacetamide can link with protein residues *via* hydrogen bonds such as ARG-230, LEU-228 (**Figure 4E**); and 1,2-Dimethylaziridine could bind to SER-227, PHE-164 from protein residues *via* hydrogen bonding (**Figure 4G**). Therefore, these four organic components from biochar could participate in N metabolism and improve the NUE in rice seedlings.

### 3.5 Biochar-extracted liquor drove the relative expression of genes involved in the nitrogen assimilation pathway

**Figure 5:**
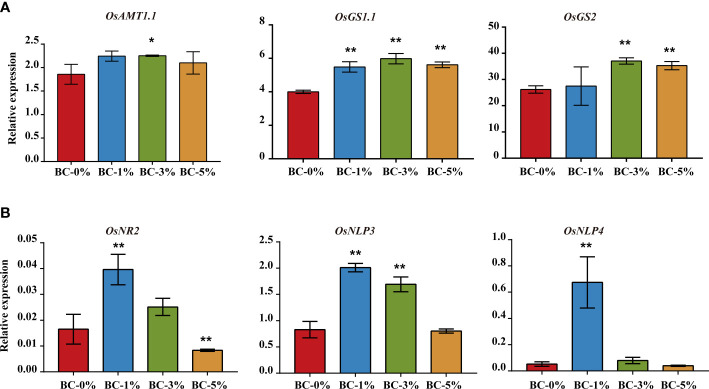
Relative expression levels of N metabolism related genes in rice seedlings. **(A)** Relative expression levels of *OsAMT1.1*, *OsGS1.1*, and *OsGS2* in leaves under ammonium N treatment. **(B)** Relative expression levels of *OsNR2*, *OsNPL3*, and *OsNPL4* in roots under nitrate N treatment. *OsActin* was used as a control, n = 3. * and ** indicate significant difference at *p* < 0.05 and *p* < 0.01, respectively. The other N metabolism related genes in rice seedlings are shown in **Figures S2**, **3**.

We demonstrated that organic molecules from biochar-extracted liquor could improve NUE in rice seedlings. In addition, the qPCR results showed that biochar-extracted liquor increased (*p* < 0.05) the expression of N metabolism related genes including *OsAMT1.1*, *OsGS1.1*, and *OsGS2* under ammonium N conditions (**Figure 5A**). Similarly, biochar-extracted liquor increased (*p* < 0.01) the expression of *OsNR2*, *OsNPL3*, and *OsNPL4* under nitrate N. These results indicate that biochar-extracted liquor promoted the expression of N metabolism-related genes and improved the NUE of rice (**Figure 5B**). In addition, we also tested the expression of some genes in the N-metabolism pathway (**Figures S2**, **S3**). We found that the biochar-extracted liquor promoted the expression of N metabolism genes. The biochar-extracted liquor significantly increased the expression of root genes (*OsNIR2*, *OsNR2*, *OsGOGAT2*, *OsAMT1.2*) and leaf genes (*OsAMT2.1*) under ${\text{NH}}_{4}^{+}‐\text{N}$ treatment. Similarly, under ${\text{NO}}_{3}^{‐}‐\text{N}$ treatment, the biochar-extracted liquor promoted the expression of root genes (*OsNRT1.1B*, *OsNIR2*, *OsSPX4*, *OsAMT1.1*) and leaf genes (*OsNRT1.1A*, *OsNR2*, *OsAMT1.1*, *OsAMT1.2*, *OsGS1.1*, *OsGS2*). On the other hand, some genes are inhibited or have no significant effect in different biochar-extracted liquor and N forms treatments, including the root genes (*OsNRT1.1A*, *OsMRT1.1B*, *OsNLP3*, *OsNLP4*, *OsSPX4*) and leaf genes (*OsNIR2*) under ${\text{NH}}_{4}{\;}^{+}-\text{N}$ treatment and the root genes (*OsNRT1.1A*, *OsGOGAT2*, *OsAMT1.2*) and leaf genes (*OsNIR2*) under ${\text{NO}}_{3}^{‐}‐\text{N}$ treatment.

**Figure 6:**
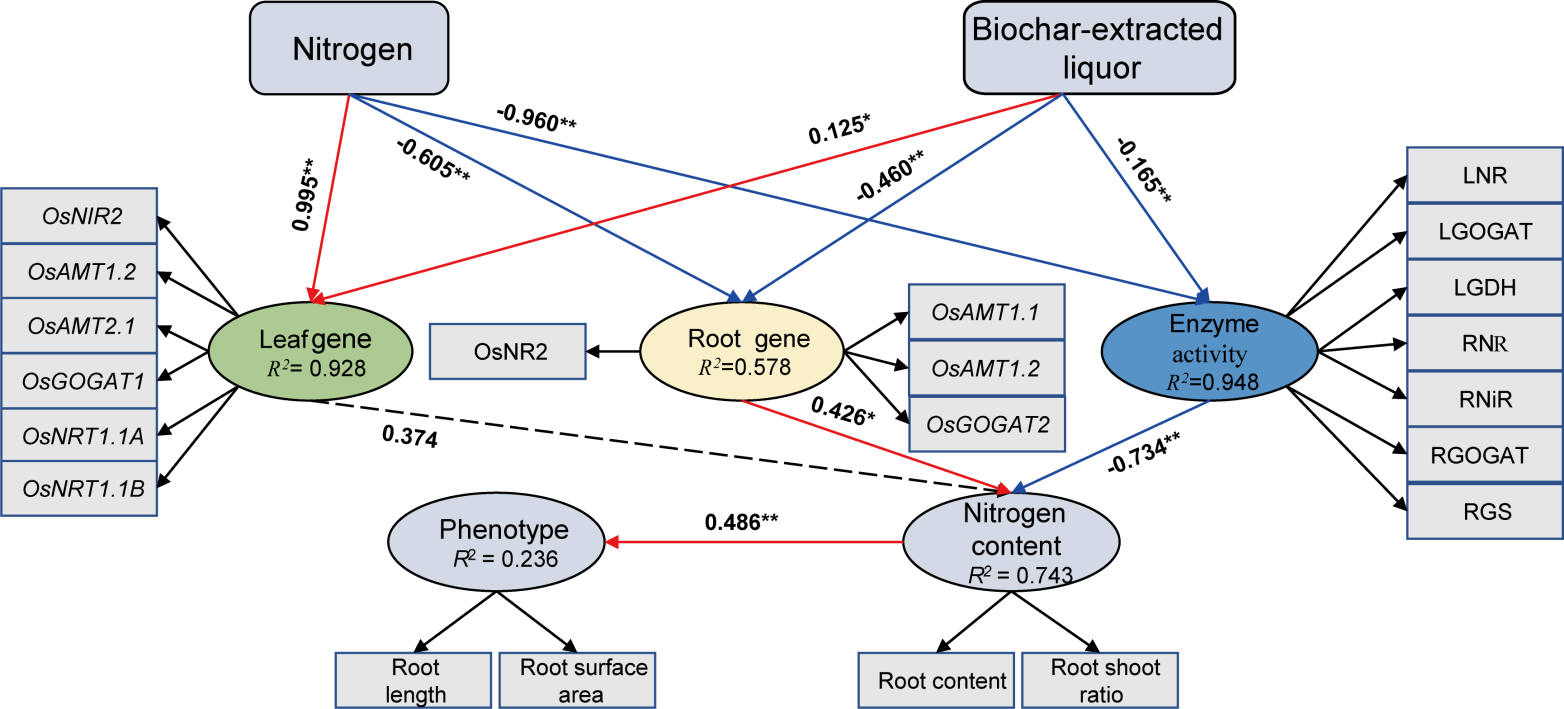
Structural equation model describing biochar-extracted liquor and N treatments affect N metabolism in rice seedlings. The path analysis numbers adjacent to arrows indicate the relationship’s effect size and the *P*-value significance. Red and blue arrows show positive and negative relationships, respectively. Paths with non-significant coefficients are presented as dotted lines. * and ** indicate significant difference at *p* < 0.05 and *p* < 0.01, respectively.

Structural equation modeling (**Figure 6**) demonstrates that the effects of biochar-extracted liquor and N addition on plant growth and N uptake, which mainly regulate N absorption of rice seedlings through leaf genes, root genes and N-metabolism enzyme activities. We found that the biochar extract could directly affect the N uptake of rice seedlings by affecting leaf genes, root genes and N metabolism enzyme activities (*p* < 0.05). N absorption of rice seedlings also actively promotes the growth of rice seedlings.

Combined with the molecular docking analysis, organic compounds including 2-Acetyl-5-methylfuran, trans-2,4-Dimethylthiane, S, S-dioxide, 2,2-Diethylacetamide, and 1,2-Dimethylaziridine from biochar-extracted liquor entered rice cell and then interacted with N-related proteins. OsAMT1.1 protein and biochar organic molecules were linked by hydrogen bonds. The interaction increased the expression of N metabolism genes and improved the NUE in rice seedlings. Therefore, testing the concentration of biochar-extracted liquor can be used as an assessment tool when evaluating the effect of biochar on plant NUE. In agricultural production, amount of biochar-added in soil for enhancing NUE should be decided by 2-Acetyl-5-methylfuran, trans-2,4-Dimethylthiane, S, S-dioxide, 2,2-Diethylacetamide, and 1,2-Dimethylaziridine concentration in biochar. Our results provide guidance for best biochar application doses.

## 4 Discussion

The findings of the present study suggested that the addition of biochar-extracted liquor (< 3%) had a positive effect. The biochar-extracted liquor treatment significantly increased rice seedling plant height, dry weight and Chlorophyll content. Prior studies reported that dilute spraying (> 300 times) of biochar extract is often considered as a bio-stimulant that stimulates root growth, biomass accumulation, nutrient uptake and nutrient mass accumulation in the soil-plant system (Lou et al., 2015; Bian et al., 2019; Gao et al., 2022; Phillips et al., 2022; Singh et al., 2022). Moreover, the increase of biochar-extracted liquor addition from 3% to 5% resulted in a decrease in plant height, biomass and chlorophyll content of rice seedlings (**Figure 1**). This indicates dose dependence for biochar-extracted liquor addition (Liu et al., 2021). Global data showed a generally negative change in crop productivity with biochar addition over 40 t ha^−1^ despite the validity of a mean increase by 10% (Liu et al., 2013).

Our results also showed that too high concentrations of biochar-extracted liquor (> 5%) had negative effects on rice seedlings growth and N uptake, while the low concentrations (< 3%) of biochar-extracted liquor was beneficial and achieved for cost-effective use of biochar. Studies have shown that the optimal dosage varies with biochar type and plant type/genotype, but most plant eco-physiological traits, such as photosynthetic capacity, chlorophyll content, and stomatal conductance, showed peak positive responses to biochar at a dosage of 2–3% (Gale and Thomas, 2019). In biochar/cropping systems, this pattern is known as the bell-shaped biochar dose/plant growth relationship, with optimal growth at moderate biochar concentrations(Jaiswal et al., 2015; Gale and Thomas, 2019; Liu et al., 2021).

Plant trait changes with biochar were often attributed to improved supply of nutrients and organic chemicals released by biochar and improved biophysical structure related to pore structure and surface functional groups of biochar (Singh et al., 2015; Ippolito et al., 2020; Lu et al., 2020; Zhang et al., 2022). Such promotion of the extract from crop residue-derived biochar was attributed to the presence of LMW organic acids and biopolymers (Bian et al., 2019), which could manipulate growth-related gene auxin binding protein and its encoded protein (Yuan et al., 2017; E et al., 2019). Thus, biochar boosting plant production and nutrient uptake in a hydroponic system could be mainly attributed to plant-promoting agents present in untreated biochar (Lou et al., 2015; Bian et al., 2019).

Organic molecules from biochar-extracted liquor are involved in the physiological and biochemical processes of plants. These organic compounds can be extracted and identified by GC-MS (Spokas et al., 2011; Yuan et al., 2017; E et al., 2019), and can perform specific functions as signal-active or hormone-like substances (Mehari et al., 2015; Bhuiya et al., 2017; Haruta and Sussman, 2017; E et al., 2020). 21 organic molecules from biochar-extracted liquor (20 polar compounds and one non-polar compound) were identified and screened by GC/MS (**Table S4**). Overall, these unstable organic molecules not only improve crop biomass and nutritional quality, but also enhance gene expression of enzymatic activities required for nutrient assimilation and protein synthesis in plants (Bian et al., 2019). The study showed that biochar-extracted liquor promoted the growth of lettuce seedlings (Oh et al., 2012). These results were postulated to be a function of the composition and concentration of the cations, anions and organic compounds in the extract (Lou et al., 2015).

Typically, biochar pyrolysis temperatures below 500°C produce unstable organic molecules (low molecular weight organic acids, phenols, aromatic hydrocarbons and alkanes) (Graber et al., 2010; Chia et al., 2014). In this study, a variety of organic compounds were present in the biochar-extracted liquor as a liquid extract of biochar by GC-MS analysis (**Table S4**). Some of these compounds, such as 6-(Methylthio)hexa-1,5-dien-3-ol and 2-acetyl-5-methylfuran, have been considered as direct biological stimulators of plant growth (Yuan et al., 2017; E et al., 2019). For example, water humus can regulate plant protein expression through energy generation, nucleic acid metabolism, carbon metabolism and some transmembrane transport in plants (Gao et al., 2015). In the present study, biochar-extracted liquor did positively and greatly promote the growth and N uptake of rice seedlings, indicating that low doses of biochar-extracted liquor did improve the metabolic activity of the plants (**Figures 1**, **2**). In addition, biochar-extracted liquor also promoted an increase in the expression of N metabolism genes (**Figures 5**, **S2**, **3**). Some low molecular weight water-soluble substances may also affect the function of ion transporters in the plasma membrane of root cells, playing a role at both the transcriptional and post transcriptional levels (Zanin et al., 2019). In addition, organic compounds of amino acid, sugars, and organic acids could be directly taken up by plants (Näsholm et al., 2009).

## 5 Conclusion

Although biochar has been widely studied in rice plant nitrogen (N) utilization, its role as a signaling factor to regulate N metabolism has rarely been reported. 21 organic molecules were identified in biochar. We found that four organic molecules were able to successfully dock and form hydrogen bonds with a N-related protein (i.e., OsAMT1.1). Biochar-extracted liquor entered plant cells and regulated the expression of N metabolism genes (*p* < 0.05), significantly increasing N uptake up to 33.60% in rice seedlings. Therefore, the combined application of N fertilizer and biochar can be a sustainable strategy to improve N uptake under controlled conditions. However, understanding the effective application of biochar in soil-plant systems, it is need to further explore the interactions between soil N cycle and plant N absorption under biochar treatment, that is, to further reveal the role of biochar in plant N metabolism. In addition, the effect of biochar on N uptake of different rice genotypes and its complex relationship with rhizosphere microbial diversity should be also the focus of future research.

## Data availability statement

The original contributions presented in the study are included in the article/**Supplementary Material**. Further inquiries can be directed to the corresponding authors.

## Author contributions

YE, JM, WC, and JG conceived of the project. SG, HW, YF, LS, and TH made a contribution to writing and editing of the manuscript. XC, DW, XZ, and HW provided constructive feedback. HC, CL, and YL supervised research and provided critical advice on the study. All authors contributed to the article and approved the submitted version.

## Supplementary material

Supplementary Figure 1 Correlation analysis of N metabolism enzymes. **(A)** Correlation analysis of enzyme activities in leaves and roots of rice seedlings treated with ammonium N; **(B)** Correlation analysis of enzyme activities in leaves and roots of rice seedlings treated with nitrate N; **(C)** Co-occurrence networks between rice phenotypic traits and enzyme activity treated with nitrate N. Phenotypic traits include plant height, root length, root surface area, aboveground weight, underground weight, root shoot ratio, root N, leaf N and chlorophyl (Chl). The N-related enzyme activity includes N metabolizing enzyme activities in leaves such as LNR, LNiR, LGOGAT, LGS, and LGDH (glutamate dehydrogenase); and roots such as RNR, RNiR, RGOGAT, and RGS. Red and blue arrows indicate the positive and negative relationships, respectively. **(D, E)** Spearman’s correlation analysis of N uptake and enzyme activity in rice seedlings under ammonium N and nitrate N forms. The confidence interval (CI) is 95%.

Supplementary Figure 2 Relative expression levels of the N metabolism related genes in rice seedlings treated with ammonium N. **(A)** Relative expression levels of the N-related genes in roots. **(B)** Relative expression levels of the N-related genes in leaves. *OsActin* was used as a control, n = 3. * and ** indicate significant difference at *p* < 0.05 and *p* < 0.01, respectively.

Supplementary Figure 3 Relative expression levels of the N metabolism related genes in rice seedlings treated with nitrate N. **(A)** Relative expression levels of the N-related genes in roots. **(B)** Relative expression levels of the N-related genes in leaves. *OsActin* was used as a control, n = 3. * and ** indicate significant difference at *p* < 0.05 and *p* < 0.01, respectively.
