# Supplementary figures and images for: Biochar-extracted liquor stimulates nitrogen related gene expression on improving nitrogen utilization in rice seedling
Source: Front Plant Sci. 2023 Jun 19;14:1131937. doi: 10.3389/fpls.2023.1131937 (PMC10317180; doi:10.3389/fpls.2023.1131937)

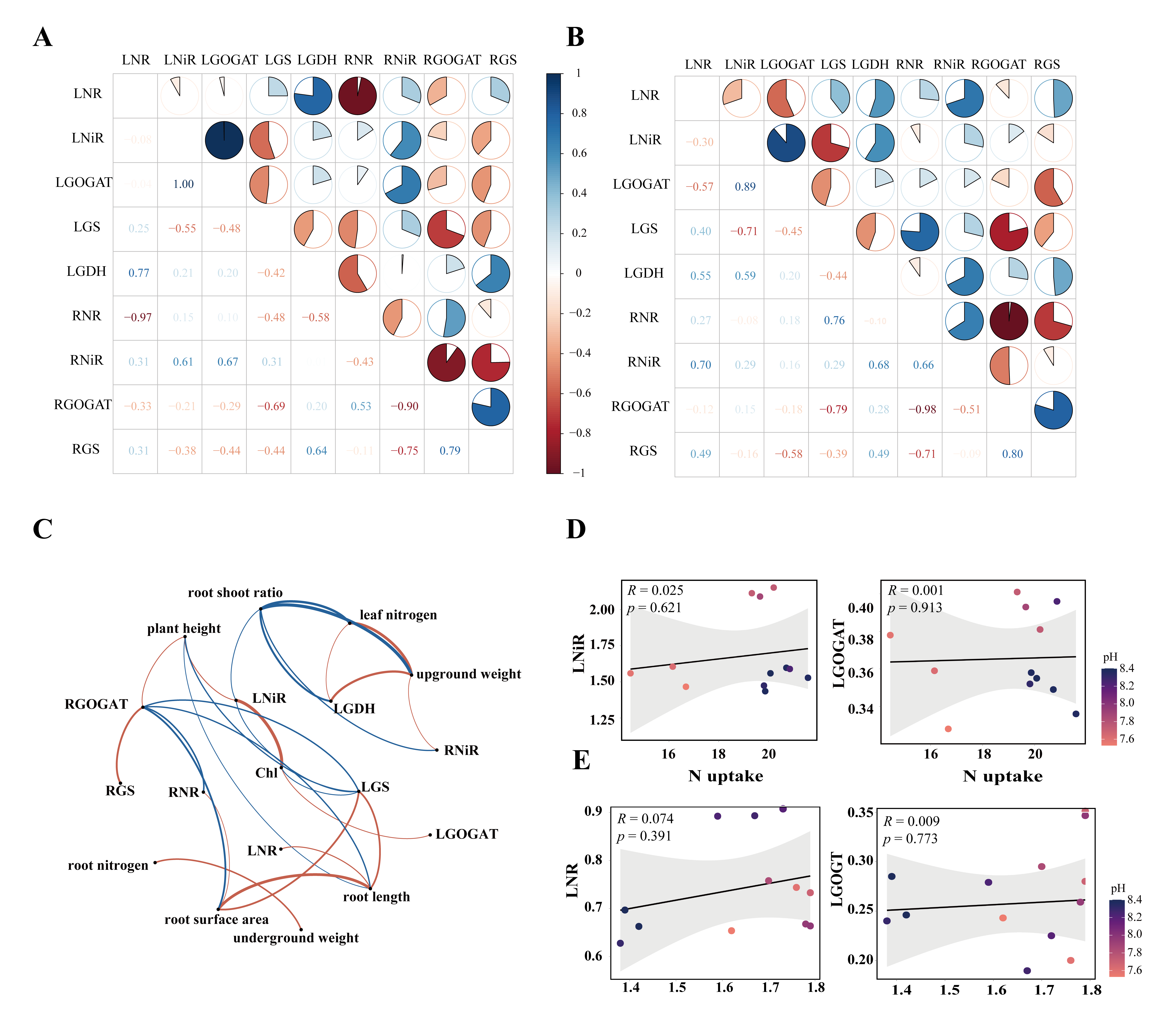

Supplement: Supplementary Figure 1 — Correlation analysis of N metabolism enzymes. (A) Correlation analysis of enzyme activities in leaves and roots of rice seedlings treated with ammonium N; (B) Correlation analysis of enzyme activities in leaves and roots of rice seedlings treated with nitrate N; (C) Co-occurrence networks between rice phenotypic traits and enzyme activity treated with nitrate N. Phenotypic traits include plant height, root length, root surface area, aboveground weight, underground weight, root shoot ratio, root N, leaf N and chlorophyl (Chl). The N-related enzyme activity includes N metabolizing enzyme activities in leaves such as LNR, LNiR, LGOGAT, LGS, and LGDH (glutamate dehydrogenase); and roots such as RNR, RNiR, RGOGAT, and RGS. Red and blue arrows indicate the positive and negative relationships, respectively. (D, E) Spearman’s correlation analysis of N uptake and enzyme activity in rice seedlings under ammonium N and nitrate N forms. The confidence interval (CI) is 95%. [file Image_1.tif]

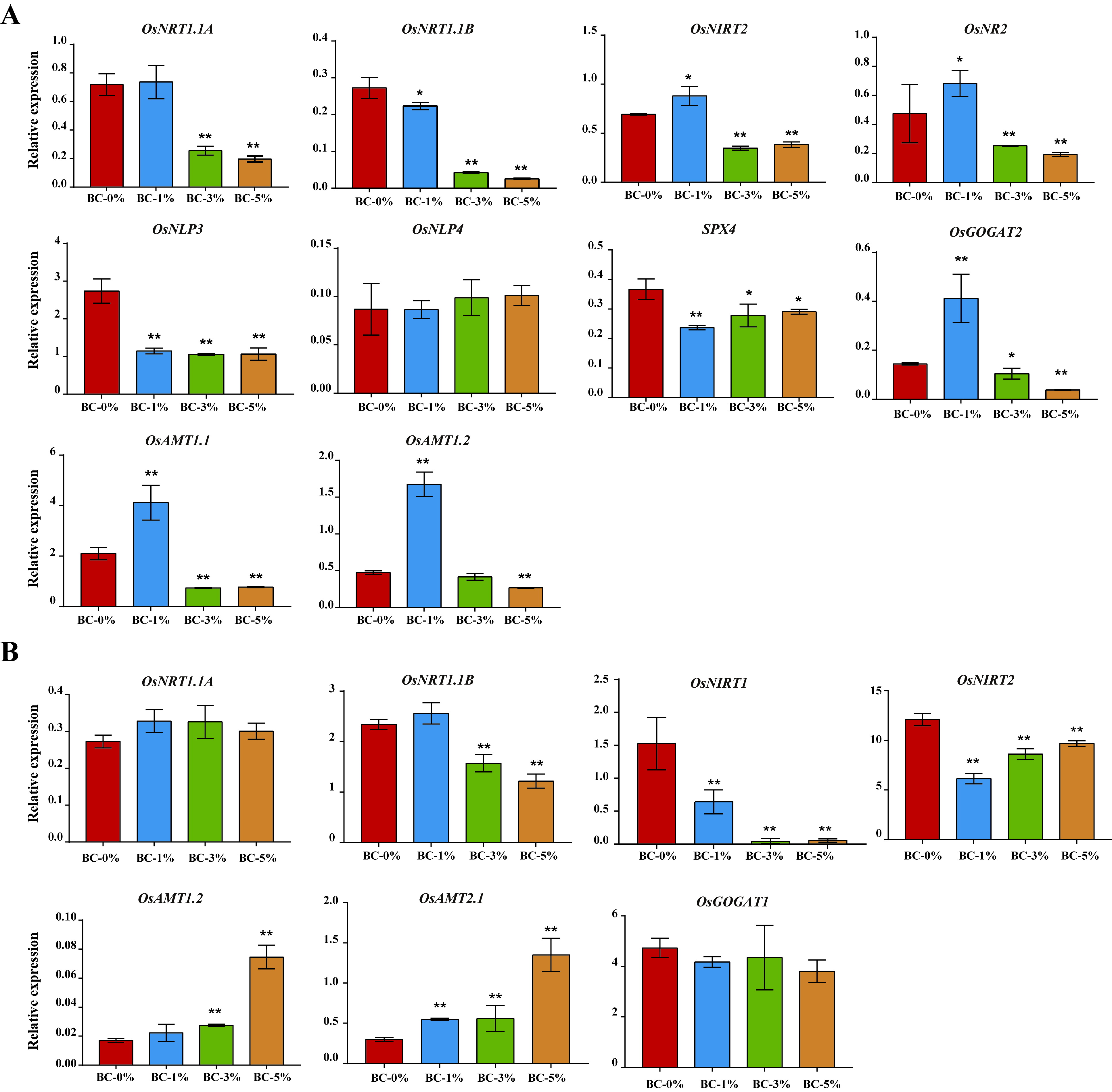

Supplement: Supplementary Figure 2 — Relative expression levels of the N metabolism related genes in rice seedlings treated with ammonium N. (A) Relative expression levels of the N-related genes in roots. (B) Relative expression levels of the N-related genes in leaves. OsActin was used as a control, n = 3. * and ** indicate significant difference at p < 0.05 and p < 0.01, respectively. [file Image_2.tif]

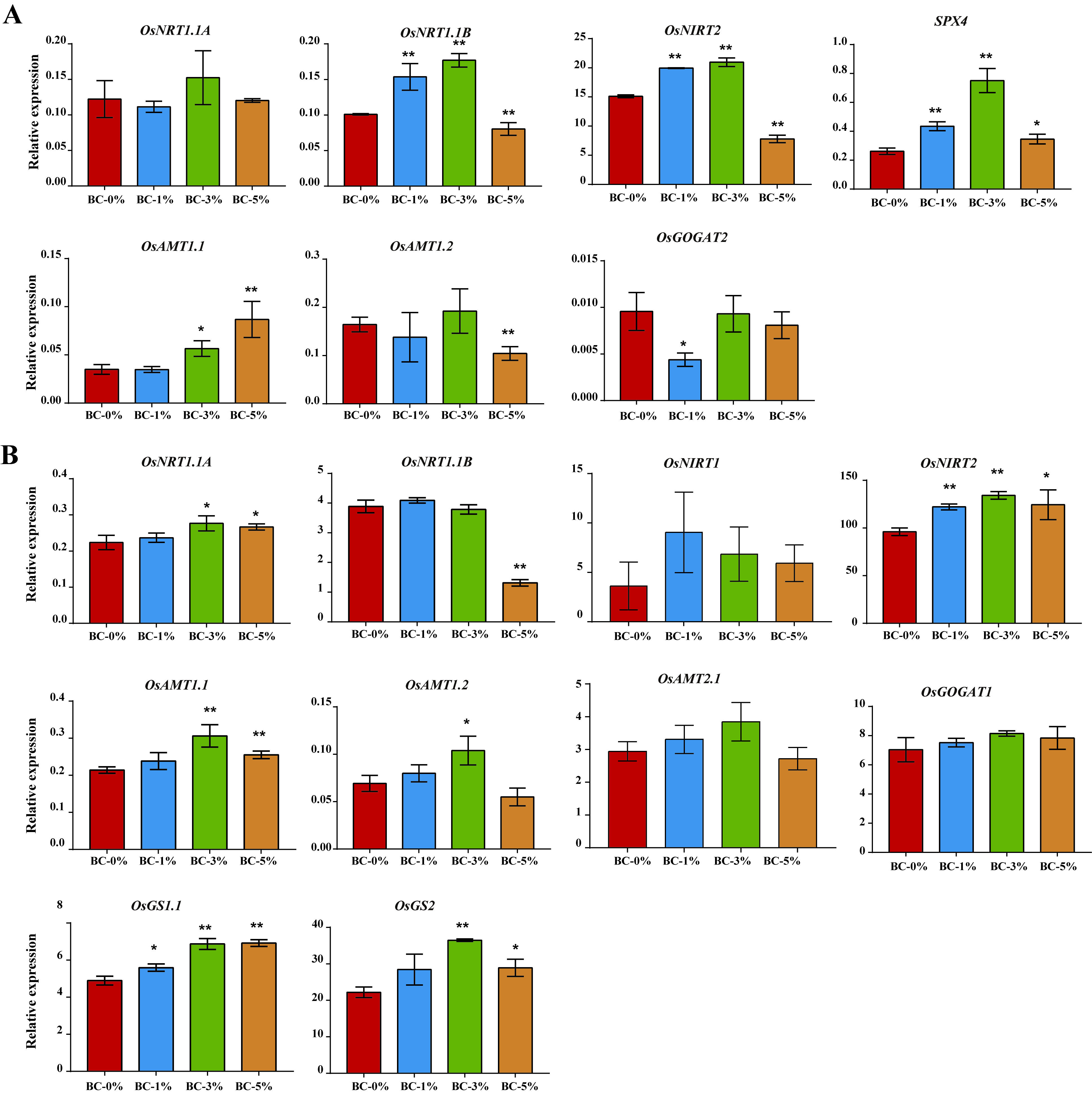

Supplement: Supplementary Figure 3 — Relative expression levels of the N metabolism related genes in rice seedlings treated with nitrate N. (A) Relative expression levels of the N-related genes in roots. (B) Relative expression levels of the N-related genes in leaves. OsActin was used as a control, n = 3. * and ** indicate significant difference at p < 0.05 and p < 0.01, respectively. [file Image_3.tif]
