# Supplementary material for: Biochar-extracted liquor stimulates nitrogen related gene expression on improving nitrogen utilization in rice seedling
Source: Front Plant Sci. 2023 Jun 19;14:1131937. doi: 10.3389/fpls.2023.1131937 (PMC10317180; doi:10.3389/fpls.2023.1131937)
Supplement: Supplementary file 4 [file Table_1.docx]

**Supplementary tables**

**Table S1** Sequences of primers required for quantitative RT-PCR

| Gene | | Forward sequence | Reverse sequence |
| --- | --- | --- | --- |
| *OsNRT1.1A* | CCCACACCAAGCAATTCAGG | | GTCTTCACCTCCTCCACGTC |
| *OsNRT1.1B* | AGGCTCGACTACTTCTACTGGC | | TGAAGAGGACGAGGTTGATGG |
| *OsNIR2* | CTGCCTCACCAAGGACAG | | TTCCTACTCCTCGTCCTCCT |
| *OsNR2* | TGGGGAGGGTGGACGAGCGGAC | | AGCGTCTCGCCGTCGCCCG |
| *SPX4* | ATCGAGAGGGAGGAGTGGTA | | AGGGAGCTATAGGTTTGCAG |
| *OsNLP3* | AGGTTGATAATTCAGGTCAGCA | | CCGTTTTGCACAAGTACATGTA |
| *OsNLP4* | AGTCGCCATTCTGACGAGAACT | | TGGGTACTCCAGTTTAGTGGAG |
| *OsAMT1.1* | GGTTTCTCTCCCTCTCCGAT | | CCACCTTCACACCACACATT |
| *OsAMT1.2* | TCGACGTACCTGCTCTTCTC | | TGTTCATGGTGTTCTTGGCG |
| *OsGS1.1* | CACCAACAAGAGGCACAATG | | ACTCCCACTGTCCTGGCAT |
| *OsGS2* | TGCTTACCCTTGACCCCAAA | | GCAAGTCATGGCGAAGTGAT |
| *OsGOGAT2* | CCTGTCGAAGGATGATGAAGGTGAAACC | | TGCATGGCCCTACTATCTTCGCATCA |
| *Actin* | TCCATCTTGGCATCTCTCAG | | GTACCCTCATCAGGCATCTG |

**Table S2** The element contents of the biochar-extracted liquor

| Elements | The concentrations of biochar-extracted liquor（mg·100 mL^-1^） | | | |
| --- | --- | --- | --- | --- |
|  |  | BC-1% | BC-3% | BC-5% |
| P |  | 0.268 | 0.656 | 1.020 |
| K |  | 0.630 | 1.519 | 2.289 |
| Na |  | 0.195 | 0.395 | 0.567 |
| Mg |  | 0.197 | 0.407 | 0.569 |
| Ca |  | 0.153 | 0.259 | 0.324 |
| B |  | 0.003 | 0.007 | 0.010 |
| Zn |  | 0.001 | 0.001 | 0.002 |
| Cu |  | 0.001 | 0.001 | 0.001 |
| Fe |  | 0.004 | 0.008 | 0.012 |
| Si |  | 1.734 | 4.137 | 5.848 |

**Table S3** General characteristics of nutrient elements in the biochar-extracted liquor

| Elements | Ammonium N treatment | |  | Nitrate N treatment | |
| --- | --- | --- | --- | --- | --- |
|  | *p* | *R*^2^ |  | *p* | *R*^2^ |
| P | 0.282 | 0.273 |  | 0.113 | 0.681 |
| K | 0.257 | 0.327 |  | 0.13 | 0.636 |
| Na | 0.223 | 0.405 |  | 0.154 | 0.573 |
| Mg | 0.214 | 0.618 |  | 0.164 | 0.548 |
| Ca | 0.229 | 0.391 |  | 0.159 | 0.561 |
| B | 0.235 | 0.377 |  | 0.147 | 0.592 |
| Zn | 0.351 | 0.133 |  | 0.122 | 0.656 |
| Cu | 0.179 | 0.511 |  | 0.201 | 0.457 |
| Fe | 0.626 | -- |  | 0.339 | 0.156 |
| Si | 0.231 | 0.386 |  | 0.153 | 0.577 |

**Table S4** The organic compounds in the biochar extracts identified by GC/MS

| Solvents | Rt (min) | Compound | Formula | Molecular weight (daltons) | Area (%) |
| --- | --- | --- | --- | --- | --- |
| Acetonitrile | 2.940 | 6-(Methylthio) hexa-1,5-dien-3-ol | C_7_H_12_OS | 144 | 1.160 |
|  | 3.650 | Formamide, N, N-diethyl- | C_5_H_11_NO | 101 | 1.455 |
|  | 3.861 | Ethanamine, N-pentylidene- | C_7_H_15_N | 113 | 2.480 |
|  | 4.814 | 2,2-Diethylacetamide | C_6_H_13_NO | 115 | 1.544 |
|  | 4.948 | Cyclopentanone, 2-(1-methylpropyl)- | C_9_H_16_O | 140 | 6.247 |
| Chloroform | 2.101 | 4,6-Dimethyl-2-thioxo-1,2-dihydro-3-pyridinecarbonitrile tbdms | C_14_H_22_N_2_SSi | 278 | 48.326 |
|  | 2.241 | Pentanamide, | C_36_H_49_N_5_O_5_ | 631 | 42.529 |
|  |  | 2-(dimethylamino)-4-methyl-N-[2-methyl-1-[[3,3a,11,12,13,14,15,15a- |  |  |  |
|  |  | octahydro-12,15-dioxo-13-(phenylmethyl)-5,8-ethenopyrrolo [3,2- |  |  |  |
|  |  | b] [1,5,8] oxadiazacyclotetradecin-1(2H)-yl] carbonyl] butyl]- |  |  |  |
|  | 4.414 | 6-(Methylthio)hexa-1,5-dien-3-ol | C_7_H_12_O_S_ | 144 | 0.146 |
|  | 5.462 | Formamide, N, N-diethyl- | C_5_H_11_NO | 101 | 0.213 |
|  | 5.757 | 1-Oxa-4-azaspiro [4.5] decan-4-oxyl, 3,3-dimethyl-8-oxo- | C_10_H_16_NO_3_ | 198 | 0.191 |
|  | 7.021 | 2-Propanamine, N, N-dimethyl- | C_5_H_13_N | 87 | 0.113 |
| Dichloromethane | 2.087 | Pyrrole[1,2-a] quinoline-1-ethanol, dodecahydro-6-(2,4-pentadienyl)-, | C_19_H_31_NO | 289 | 49.163 |
|  |  | [1R-[1. alpha.,3a. beta.,5a. alpha.,6. alpha. (Z),9a.alpha.]]- |  |  |  |
|  | 4.523 | 6-(Methylthio) hexa-1,5-dien-3-ol | C_7_H_12_OS | 144 | 0.118 |
|  | 5.759 | Formamide, N,N-diethyl- | C_5_H_11_NO | 101 | 0.177 |
|  | 5.925 | Ethanamine, N-pentylidene- | C_7_H_15_N | 113 | 0.170 |
|  | 6.666 | trans-2,4-Dimethylthiane, S, S-dioxide | C_7_H_14_O2S | 162 | 0.076 |
|  | 7.022 | Acetamide, N, N-diethyl- | C_6_H_13_NO | 115 | 0.111 |
|  | 7.158 | 2,2-Diethylacetamide | C_6_H_13_NO | 115 | 0.040 |
|  | 7.821 | Cyclopentanone, 2-(1-methylpropyl)- | C_9_H_16_O | 140 | 0.014 |
|  | 16.096 | 2-Acetyl-5-methylfuran | C_7_H_8_O_2_ | 124 | 0.018 |
|  | 18.441 | Tritetracontane | C_43_H_88_ | 604 | 0.012 |
| Ethyl acetate | 2.932 | 6-(Methylthio) hexa-1,5-dien-3-ol | C_7_H_12_OS | 144 | 2.424 |
|  | 3.649 | Formamide, N, N-diethyl- | C_5_H_11_NO | 101 | 2.005 |
|  | 4.529 | Acetamide, N,N-diethyl- | C_6_H_13_NO | 115 | 1.089 |
| Ethyl alcohol | 2.076 | (1R,2R,4S)-2-(6-Chloropyridin-3-yl)-7-azabicyclo [2.2.1] heptane | C_11_H_13_CIN_2_ | 208 | 1.556 |
|  | 3.149 | 6-(Methylthio)hexa-1,5-dien-3-ol | C_7_H_12_OS | 144 | 1.871 |
|  | 3.938 | Formamide, N, N-diethyl- | C_5_H_11_NO | 101 | 1.725 |
|  | 4.169 | 1,2-Dimethylaziridine | C_4_H_9_N | 71 | 2.151 |
|  | 5.198 | Pyrrole, 2-(4-methyl-5-cis-phenyl-1,3-oxazolidin-2-yl)- | C_14_H_16_N_2_O | 228 | 1.027 |
| Methyl alcohol | 2.124 | Pentanamide, | C_36_H_49_N_5_O_5_ | 631 | 79.126 |
|  |  | 2-(dimethylamino)-4-methyl-N- [2-methyl-1-[[3,3a,11,12,13,14,15,15a- |  |  |  |
|  |  | octahydro-12,15-dioxo-13-(phenylmethyl)-5,8-ethenopyrrolo [3,2- |  |  |  |
|  |  | b] [1,5,8] oxadiazacyclotetradecin-1(2H)-yl]carbonyl]butyl]- |  |  |  |
|  | 2.225 | Pyrrolidin-2-one, 1-[1-(4-carbomethoxyphenyl) butan-1-ol-2-yl]- | C_15_H_12_Cl_2_O_3_ | 310 | 0.230 |
|  | 5.741 | Ethanamine, N-pentylidene- | C_7_H_15_N | 113 | 0.116 |
| n-hexane | 2.057 | Cyclopentane, 1,2,3-trimethyl- | C_8_H_16_ | 112 | 100 |
| n-heptane | – | – | – | – | – |
